# The many lives of Myc in the pancreatic β-cell

**DOI:** 10.1074/jbc.REV120.011149

**Published:** 2020-12-02

**Authors:** Carolina Rosselot, Sharon Baumel-Alterzon, Yansui Li, Gabriel Brill, Luca Lambertini, Liora S. Katz, Geming Lu, Adolfo Garcia-Ocaña, Donald K. Scott

**Affiliations:** Diabetes Obesity Metabolism Institute, and the Mindich Child Health and Development Institute, The Icahn School of Medicine at Mount Sinai, New York, New York, USA

**Keywords:** Myc, pancreatic β-cell, glucose, diabetes, proliferation, adaptation, aging, DNA methylation, bHLHZ, basic helix–loop–helix–leucine zipper, ChoREs, carbohydrate response elements, ChREBP, carbohydrate response element binding protein, CDKs, cyclin-dependent kinases, GSIS, glucose-stimulated insulin secretion, HFD, high-fat diet, lncRNA, long noncoding RNA, IRES, internal ribosomal entry site, mTOR, mammalian target of rapamycin, MAOs, monoamine oxidases, NADPH, nicotinamide adenine dinucleotide phosphate, NLS, nuclear localization signal, ncRNAs, noncoding RNAs, PVT1, plasmacytoma variant translocation 1, p-TEFb, positive transcription elongation factor b, PKC ζ, protein kinase C ζ, ROS, reactive oxygen species, T1D, type 1 diabetes, T2D, type 2 diabetes, TAD, transcriptional activation domain, TRAAP, transformation/transcription domain-associated protein

## Abstract

Diabetes results from insufficient numbers of functional pancreatic β-cells. Thus, increasing the number of available functional β-cells *ex vivo* for transplantation, or regenerating them *in situ* in diabetic patients, is a major focus of diabetes research. The transcription factor, Myc, discovered decades ago lies at the nexus of most, if not all, known proliferative pathways. Based on this, many studies in the 1990s and early 2000s explored the potential of harnessing Myc expression to expand β-cells for diabetes treatment. Nearly all these studies in β-cells used pathophysiological or supraphysiological levels of Myc and reported enhanced β-cell death, dedifferentiation, or the formation of insulinomas if cooverexpressed with Bcl-xL, an inhibitor of apoptosis. This obviously reduced the enthusiasm for Myc as a therapeutic target for β-cell regeneration. However, recent studies indicate that “gentle” induction of Myc expression enhances β-cell replication without induction of cell death or loss of insulin secretion, suggesting that appropriate levels of Myc could have therapeutic potential for β-cell regeneration. Furthermore, although it has been known for decades that Myc is induced by glucose in β-cells, very little is known about how this essential anabolic transcription factor perceives and responds to nutrients and increased insulin demand *in vivo*. Here we summarize the previous and recent knowledge of Myc in the β-cell, its potential for β-cell regeneration, and its physiological importance for neonatal and adaptive β-cell expansion.

Diabetes is a chronic disease that occurs when the body is unable to process blood glucose properly. Insulin is the hormone that regulates the cellular uptake of glucose to be used for energy in the cell. Pancreatic β-cells are unique in their ability to secrete insulin in response to a rise in plasma glucose, and insufficient insulin secretion from β-cells leads to the development of diabetes. This insulin secretion insufficiency occurs when there is an absolute (Type 1 diabetes, T1D) or relative (Type 2 diabetes, T2D) decrease in the number of β-cells (1, 2). In mammals, the number of β-cells required to maintain proper glucose homeostasis reflects a dynamic balance between cell growth and apoptosis. Patients with either T1D or T2D would benefit from therapies that protect and expand functional β-cell mass (3, 4). Thus, increasing the number of available β-cells by expanding functional β-cell mass *ex vivo* for transplantation, or *in vivo* in diabetic patients, is one of the priorities in diabetes research (Fig. 1).

**Figure 1:**
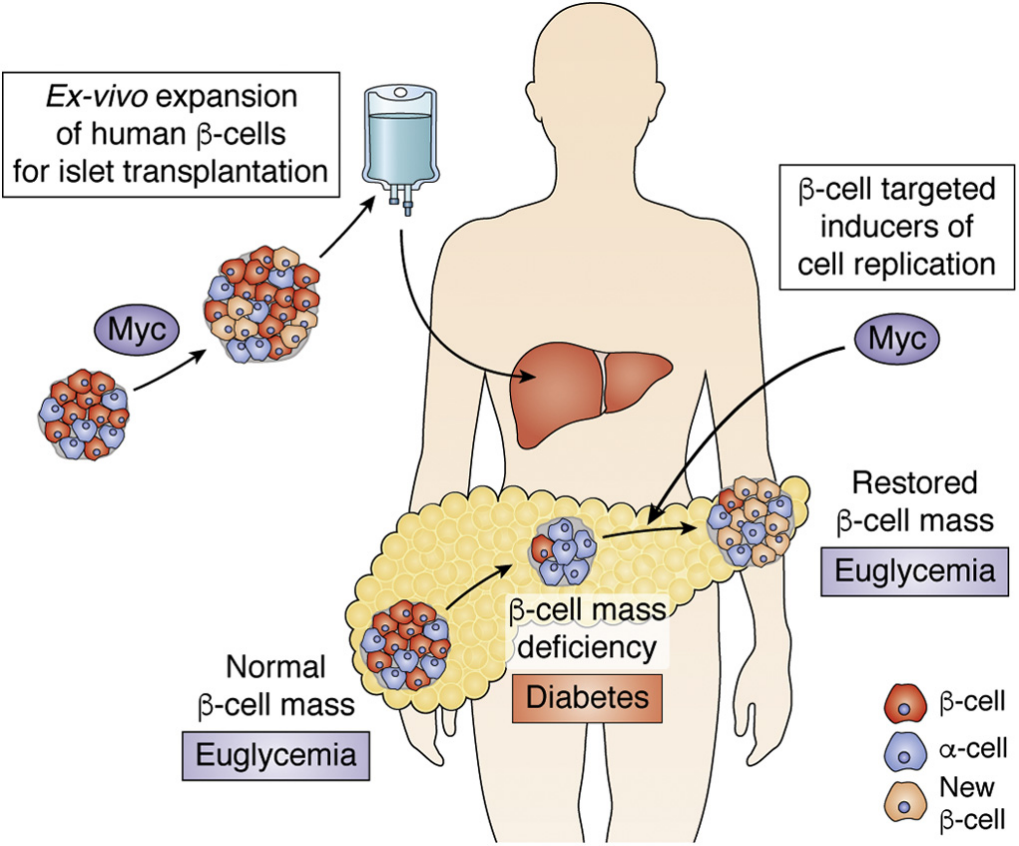
**Strategies to increase beta cell mass in diabetes.** Diabetes occurs when there is a deficiency in functional β-cell mass. β-cell regeneration could be achieved by increasing β-cell replication using Myc-based β-cell-targeted agents. Alternatively, these Myc-based agents could be used to expand β-cells *ex vivo* for transplantation.

During postnatal development, β-cells are highly proliferative and their expansion contributes to a substantial increase in β-cell mass (3, 4, 5, 6). With advancing age, the rate of β-cell proliferation in rodents and humans diminishes dramatically (5, 6). Compensatory β-cell proliferation and mass occurs at the onset of increased physiological and metabolic insulin demand (7, 8, 9, 10). β-cell dysfunction and absence of compensatory expansion of β-cell mass, followed by loss of β-cells due to apoptosis, are the ultimate events leading to the development of T2D (2, 11, 12). β-cell death is induced by multiple stressors such as glucotoxicity, lipotoxicity, proinflammatory cytokines, endoplasmic reticulum stress, and oxidative stress (13, 14, 15, 16, 17, 18). The initial trigger and orchestration of events that lead to the initiation of β-cell death remain unclear. Therefore, the identification of key regulators of β-cell death during chronic hyperglycemia and hyperlipidemia would offer novel therapeutic targets for the treatment of diabetes.

The transcription factor Myc regulates the expression of genes involved in cell growth, proliferation, apoptosis, organellogenesis, and metabolism (19, 20, 21). Myc is normally expressed at very low levels in β-cells and can be induced by glucose, a β-cell mitogen (22, 23, 24, 25). These observations posed two questions: (1) is Myc a key regulator of β-cell death in chronic hyperglycemia? and (2) is Myc capable of driving therapeutic β-cell proliferation? To answer these questions, several groups generated transgenic mice with constitutive or inducible overexpression of Myc in the β-cell (26, 27, 28, 29). Transgenic mice expressing very high levels of Myc in β-cells (estimated in the 20- to 50-fold range) (30) display increased β-cell proliferation and apoptosis, downregulation of insulin gene expression, and development of diabetes. Thus, Myc is a likely contributor to glucose toxicity when its expression is sustained at very high levels in β-cells. These studies depicted Myc upregulation as a negative event in the β-cell that could lead to cell destruction and diabetes, dimming the idea of harnessing Myc expression to expand β-cell mass for diabetes. Studies in the last decade, on the other hand, have demonstrated that “gentle” induction of Myc expression in rodent and human β-cells enhances β-cell replication without induction of cell death or loss of insulin secretion, suggesting that appropriate levels of Myc could have therapeutic potential for β-cell regeneration (30, 31).

Critically, the normal physiological role of Myc in β-cell biology is barely known. Two recent studies using β-cell specific Myc knockout mice have provided the first *in vivo* evidence indicating that Myc plays a crucial role in the growth and function of the β-cell and that the destructive nature of Myc only develops after prolonged metabolic insult resulting in inappropriate chronic high levels of expression (32, 33) (Fig. 2). The first study demonstrated that (i) Myc is required for postnatal β-cell proliferation and (ii) that mild, lifelong Myc overexpression in the mouse β-cell markedly enhances β-cell mass and leads to sustained mild hypoglycemia, without induction of tumorigenesis (32). The second study reported that in response to a 1-week hypercaloric diet, Myc protein levels increase in mouse β-cells independent of age and that Myc is necessary for the normal adaptive β-cell expansion that occurs in young mice (33). In total, these unique recent data and the available literature provide strong support for the idea that Myc is crucial for potential β-cell regenerative approaches as well as for the normal physiology of the β-cell under basal or metabolically stressed conditions. The role of Myc in β-cells is reviewed from this point of view in the sections below.

**Figure 2:**
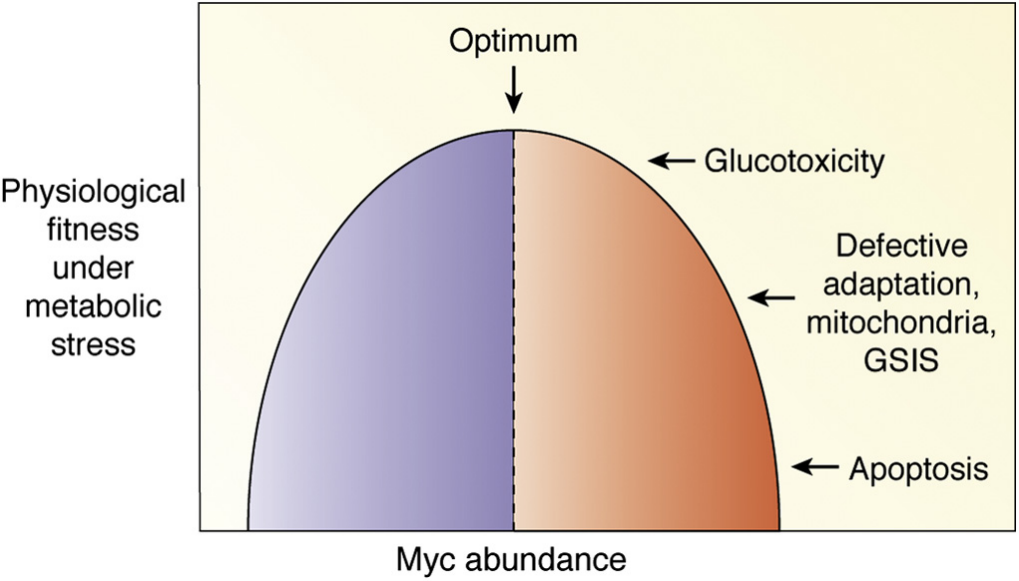
**Bell-shaped curve for Myc in β-cells under metabolic stress.** Most of what is known about Myc in β-cells is from either supraphysiologic or pathophysiologic concentrations of Myc, resulting in glucotoxicity or apoptosis (*shaded*). By contrast, little is known about Myc in its native context including whether Myc is necessary for normal adaptive proliferation, mitochondrial activity, and glucose-stimulated insulin secretion (GSIS, *unshaded*). All these points are examined in this review.

## The Myc transcription factor: functions, structure, and regulation

c-Myc (also referred to as Myc) was originally discovered in the late 1970s after researchers revealed the homology between an oncogene carried by the Avian Myelocytomatosis virus and a human gene overexpressed in various cancers (34). Later discovery of closely homologous genes in humans led to the addition of n-Myc and l-Myc to this family of regulator genes and proto-oncogenes that code for transcription factors (34, 35).

**Figure 3:**
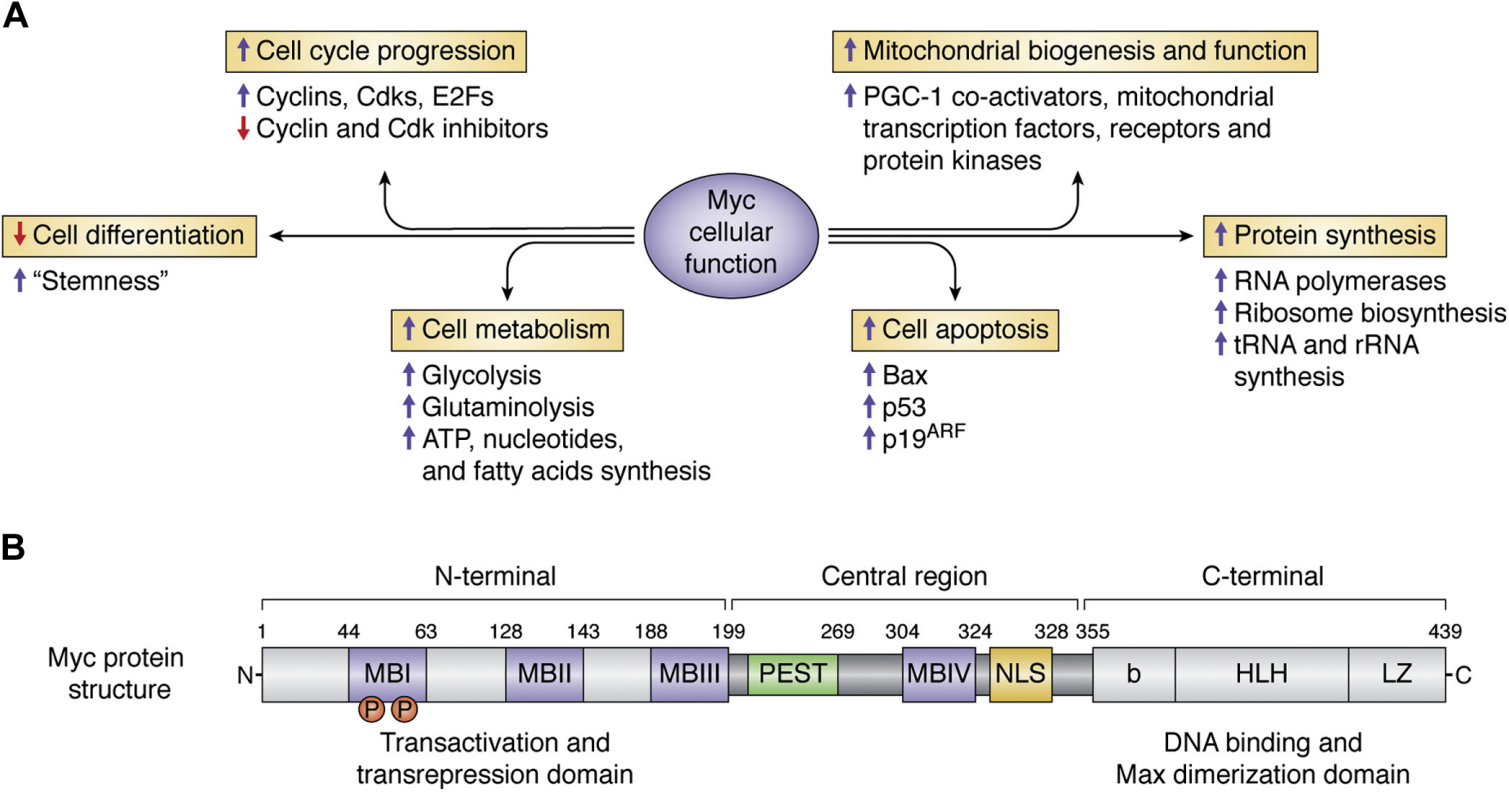
**Functions and structure of Myc**. *A*, Myc regulates multiple biological actions essential for the expansion, survival, and normal function of the cell including cell cycle progression and proliferation, cell apoptosis, cell differentiation, cell metabolism, protein synthesis, and mitochondrial biogenesis and function. *B*, the structure of the Myc protein is highly complex and composed of three regions (N-terminal, central, and C-terminal) containing several domains that are essential for transactivation (Myc Box (MB) I and II), transrepression (MBIII), apoptosis (MBIV), nuclear transport (nuclear localization signal NLS), and DNA binding and Max dimerization (basic (b), helix–loop–helix (HLH) and leucine zipper domain (LZ)). The PEST domain is a polypeptide sequence rich in proline (P), glutamic acid (E), serine (S), and threonine (T). Myc gets phosphorylated at Thr58 and Ser62 and that affects its stability.

It is obvious from its discovery that most of researchers' attention has focused on Myc's ability to promote cell growth and proliferation by stimulation of cell cycle progression (36). Accordingly, Myc increases the expression of multiple cyclins, cyclin-dependent kinases (CDKs), and E2F transcription factors, while decreasing the expression of cell cycle inhibitors (37, 38). It is therefore not surprising that Myc is an important prognostic factor in many types of aggressive cancers (39, 40, 41, 42, 43, 44). Yet, over the years it became clear that this moonlighting protein controls multiple distinct functions within the cell (Fig. 3*A*). One example is that Myc attenuates the differentiation of numerous cell types during development, thus preserving the “stemness” of these cells (45). Moreover, although being associated with cell division, Myc expression promotes apoptosis when growth factors are limiting (46). Another example is that Myc strongly influences cell metabolism. Myc expression stimulates the glycolysis and glutaminolysis pathways, both of which promote cell proliferation by increasing the synthesis of ATP, nucleotides, and fatty acids that serve as building blocks for dividing cells (47, 48, 49). Myc also induces mitochondrial biogenesis and increases mitochondrial function through activation of PGC-1 coactivators, mitochondrial transcription factors, mitochondrial receptors, and protein kinases (48, 49). Myc participates in the stimulation of global protein expression for the purpose of increasing cell mass before cell division, through the activation of RNA polymerase I, II, and III and of genes that take part in ribosome biosynthesis, ribosome structure, and tRNA and rRNA synthesis (47, 49). Therefore, Myc carries out many biological actions essential for the expansion, survival, and normal function of the cell. Consequently, modifications in Myc's expression, sequence, or structure can lead to altered cellular behavior resulting in pathologies ranging from mild dysfunction, to tumorigenesis, and even to cell death.

With so many distinct functions within the cell, the structure of the Myc protein is highly complex and composed of several domains that are essential for its activity (19, 35) (Fig. 3*B*). At the N-terminal region, Myc contains a transcriptional activation domain (TAD) that together with the Myc boxes, MBI and MBII, is necessary for Myc's transcriptional and cell transforming activity. Additionally, Myc contains an MBIII region that is responsible for Myc's transcriptional repression activity. The central region contains a nuclear localization signal (NLS) and a MBIV box that is necessary for both Myc's transcriptional activity and apoptotic signaling. The Myc C-terminal region is composed of a basic domain, which enables the Myc DNA binding activity, and a leucine zipper domain that is necessary for Myc binding to its obligate heterodimer partner, Max. Once the Myc–Max complex is formed, Myc binds to E-box sequences (CAC(G/A)TG) and stimulation of transcription at the promoter-proximal E box occurs (35, 50). Since the E-box is only 6 bp, it occurs with a high random frequency in the genome. Accordingly, there are many thousands of binding sites for Myc, and since there are numerous other transcription factors that recognize E-boxes, there is an inherent competition with Myc for DNA binding (50). In the β-cell, one such transcription factor that is upregulated in response to glucose and that can bind to some E-box elements is carbohydrate response element binding protein (ChREBP) (51). In response to increased glucose metabolism, ChREBP binds to carbohydrate response elements (ChoREs), which are composed of 2 E-boxes (or sequences closely resembling E-boxes), separated by 5 bp (52). Thus, one might predict that in the context of double E-boxes separated by 5 bp, there may be circumstances where Myc and ChREBP compete for binding to the same site, and only one transcription factor remains bound to a particular regulatory locus. However, in β-cells, both Myc and ChREBP are recruited to glucose-responsive target genes at the same time (53). This is because Myc can interact with components of the transcriptional machinery, such as transformation/transcription domain-associated protein (TRAAP) and positive transcription elongation factor b (p-TEFb) that regulate transcriptional initiation and elongation, respectively, and without necessarily binding DNA (54, 55). In fact, in cancer cells, overexpression of Myc acts as an amplifier of essentially all genes that are active in that cell at the moment of overexpression, and Myc retains transformation activity even after deletion of its DNA binding domain (56, 57). In a more physiological context, in β-cells, Myc increases about 1.5–3-fold after exposure to increased glucose (22, 23). Using a chromatin immunoprecipitation assay, and multiple primers across the promoter and transcription start site (TSS) of a prototypical glucose-responsive gene, *Pklr*, ChREBP is recruited specifically to the carbohydrate response element, with a narrow peak centered over the ChoRE, about 200 bp upstream of the TSS. At the same time, Myc is recruited to the same genomic region, but with a broad peak, starting upstream of the ChoRE and extending nearly 1000 bp downstream of the transcription start site (53). Importantly, there is no consensus E-box in this region of DNA, suggesting that in this case, Myc is not interacting directly with DNA. In addition, Myc activity is necessary for the recruitment of ChREBP to DNA, so that knockdown of Myc with siRNA, or a chemical inhibitor of Myc, blocks the ability of ChREBP to bind to its cognate response element (53, 58, 59). Thus, Myc and ChREBP cooperate to mediate a glucose-responsive gene expression in β-cells.

Since changes in Myc expression can result in important functional outcomes for the cell, Myc levels are tightly controlled by a sophisticated regulatory network. At the transcriptional level, *Myc* expression is controlled by four different promoters and over 30 transcription factors from multiple regulatory pathways (60). At the translational level, the 5ʹ untranslated region of *Myc* mRNA is highly structured and contains an internal ribosomal entry site (IRES) that allows regulation of *Myc* translation during development and in response to genotoxic stress (61). At the protein level, Myc stability is controlled by multiple ubiquitin ligases, resulting in extremely short half-life of around 20–30 min (62). Moreover, posttranscriptional modifications, such as phosphorylation, ubiquitination, and acetylation, regulate Myc stability and function (63), and Myc transcriptional activity is negatively controlled by a short Myc variant called “Myc-nick” during a variety of stresses (64). Importantly, although Myc does not dimerize with other basic helix–loop–helix–leucine zipper (bHLHZ) proteins other than Max, Max dimerizes with other bHLHZ proteins such as the Mxd family of proteins and Mga. The multiple interactions of Max and bHLHZ proteins appear to form an extended network through which Myc mediates a broad transcriptional response to mitogenic, growth arrest, and metabolic signals (65). Myc also partners with the adjacent long noncoding RNA (lncRNA) plasmacytoma variant translocation 1 (PVT1), which stabilizes Myc protein and potentiates its activity (66). On the other hand, a recent study demonstrated that PVT1 can also act as a tumor suppressor (67); thus, PVT1 can either promote or inhibit Myc activity depending on cellular context. In summary, multiple regulatory aspects control the expression levels of Myc due to its high relevance for the life of the cell. Additional details on the regulation, cellular functions, structure, and biology of Myc have been described over the years in excellent reviews, and we refer the reader to those publications for additional knowledge on these aspects of Myc (19, 20, 21, 35, 37, 38, 39, 42, 45, 46, 47, 49, 62, 63, 65, 66, 67, 68).

The *Myc* promoter binds a multitude of transcription factors, which act as relay switches of a large variety of signal transduction pathways integrating multiple cellular signals and mediating a transcriptional response that drives cell growth and proliferation and impacts differentiation, survival, and pluripotency (69). The signal transduction pathways may be initiated by hormones, growth factors, changes in metabolism, or any of a number of perceived changes in the environment, such as oxygen tension in the liver or mechanical loading in the muscle (59, 69, 70, 71). The induction of Myc then drives the expression of other transcription factors, which may then bind the *Myc* promoter to either accelerate or repress its activity. In this manner, the *Myc* promoter is connected to, regulates, and is regulated by, many feedback networks (69). Thus, the transcriptional regulation of the *Myc* gene, and its subsequent regulation at the mRNA and protein levels by multiple environmental cues, constitutes a crucial cellular sensor that provides the cell with information required to proceed with critical functional decisions such as cellular growth, division, or cell death. Because of its important nexus in pathophysiology, research efforts have been more recently focused on elucidating Myc regulation during cellular stress (64, 68, 72). In the diabetes field, since constant hyperglycemia leads to initial compensatory β-cell growth followed by functional decompensation and death, Myc regulation in this scenario has been thoroughly studied as discussed in the next sections.

## Glucose regulates Myc expression in the pancreatic β-cell

### Glucose-mediated regulation of Myc expression in β-cells *in vitro*

In 1988, Yamashita *et al*. (73) reported for the first time that glucose rapidly increases *Myc* mRNA expression in the rat insulinoma cell line, RINr, in a dose-response fashion. RINr cell proliferation is dramatically increased after 24 h of glucose addition, suggesting that glucose-induced proliferation of RINr cells associates with the stimulation of *Myc* gene expression. This finding was reproduced in another set of experiments using primary adult rodent islets where *Myc* expression is normally very low (30, 74). Accordingly, *Myc* mRNA expression is maximally increased at 18 h of incubation with high concentrations of glucose (74, 75, 76). Similarly, incubation of rat insulinoma-derived INS-1832/13 cells with high glucose increases *Myc* gene expression two- to sevenfold (53, 59). Importantly, Myc protein expression increases two- to threefold in primary rat and mouse islets exposed to high glucose for 24 h (33, 74). In summary, glucose rapidly stimulates *Myc* mRNA and Myc protein expression in β-cells *in vitro*, which has potential relevance in hyperglycemic situations *in vivo*.

**Figure 4:**
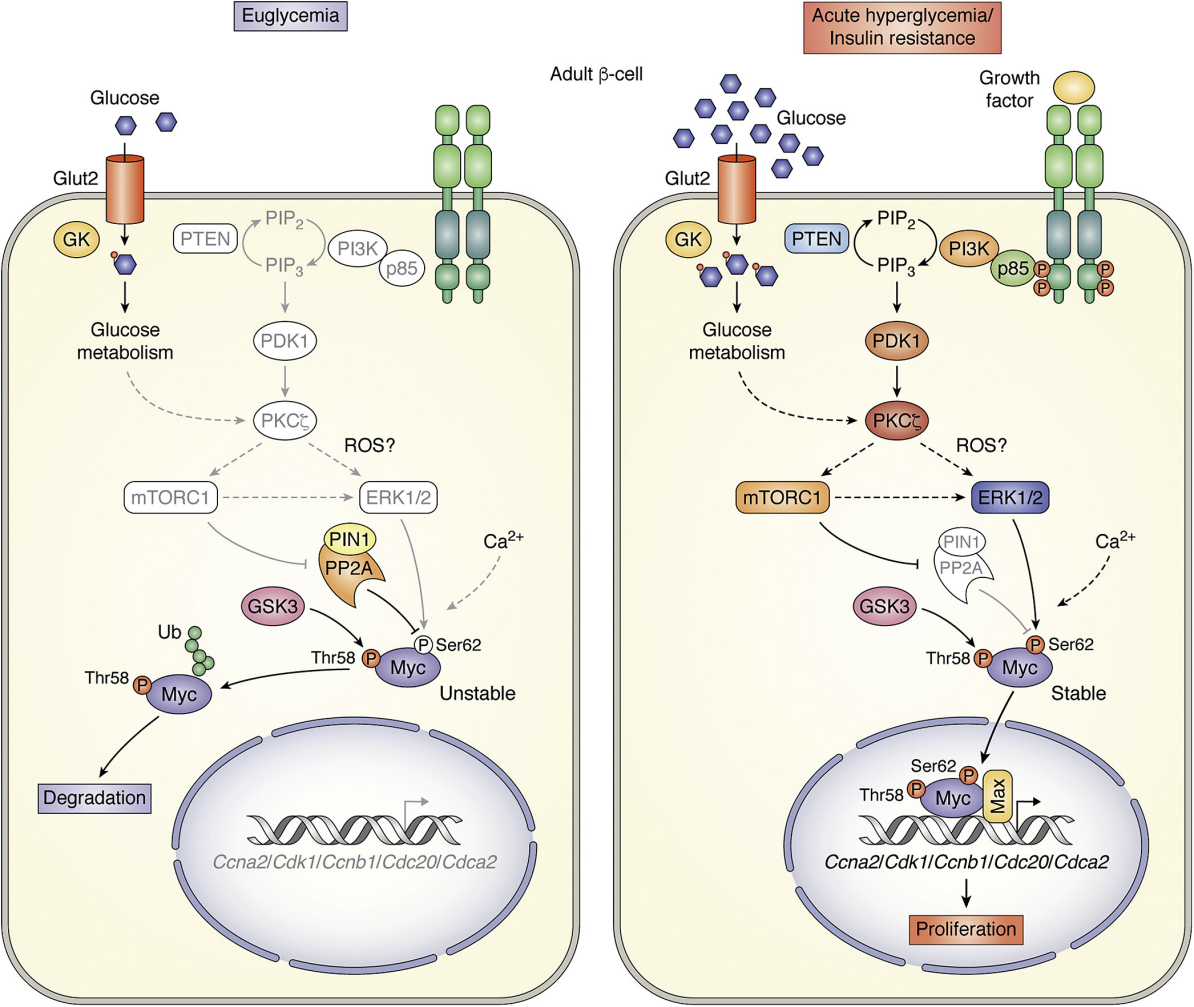
**Signaling pathways that regulate Myc protein levels in β-cells in basal conditions and in situations of hyperglycemia and increased insulin demand.** Signaling pathways depicted here have been inferred from studies in references 33, 74–84 and, 100. Dashed arrows indicate potential pathways. Glucose transporter 2 (Glut2) facilitates glucose movement across the cell membrane. Glycogen synthase kinase 3 (GSK3) phosphorylates Myc on Thr58. In basal conditions (euglycemia), the phosphatase PP2A is not repressed by mTORC1, which leads to Ser62-Myc dephosphorylation, decreased Myc stability, and degradation in β-cells. In acute hyperglycemic events and when insulin demand is increased, PI3K is activated leading to conversion of PIP2 to PIP3 (an action that can be reversed by PTEN), which localizes PDK1 close to the plasma membrane where PDK1-mediated activation of PKC ζ occurs. This leads to mTORC1 activation that impairs PP2A activity preserving Myc phosphorylation on Ser62 by ERK1/2 and increasing Myc stability in β-cells. Myc then translocates to the nucleus with its partner Max, binds to E-boxes on promoters of cell cycle genes such as cyclin A2 (*Ccna2*), cyclin-dependent kinase 1 (*Cdk1*), cyclin B1 (*Ccnb1*), cell division cycle protein 20 (*Cdc20*), and cell division cycle associated 2 (*Cdca2*), and induces adaptive β-cell proliferation.

A variety of Myc regulatory mechanisms in β-cells have been reported. Jonas *et al*. (74) showed that glucose stimulates Myc expression in rodent islets by increasing cytosolic calcium and cAMP levels. Although other studies have also reported calcium as a signal for increasing Myc levels (77), the role of cAMP in regulating Myc expression remains controversial, since evidence for both negative and positive regulation of Myc expression has been reported (78, 79, 80). Elouil *et al*. (75) demonstrated that addition of the antioxidant N-acetyl cysteine (NAC) to primary rat islets blocks both hydrogen peroxide and glucose-stimulated Myc upregulation, suggesting that high glucose stimulates Myc expression in pancreatic islets through the generation of reactive oxygen species (ROS). A similar phenomenon is found in other cell types, where incubation of cells with hydrogen peroxide increases Myc expression (81, 82). Nonetheless, this regulatory mechanism of Myc expression has not been studied in detail in β-cells. Studies on human melanoma cells suggest that ROS formation promotes ERK-dependent Myc phosphorylation at Ser62, which stabilizes Myc protein (83). In high glucose, protein kinase C ζ (PKC ζ) is necessary for the generation of ROS by nicotinamide adenine dinucleotide phosphate (NADPH) oxidase (84), and it is therefore not surprising that PKC ζ activation increases Myc protein stability at high glucose in mouse β-cells (33). PKC ζ is necessary for glucose-stimulated ERK1/2-dependent Myc Ser62 phosphorylation and for the mammalian target of rapamycin (mTOR)-dependent decrease of PP2A phosphatase activity enhancing Myc protein stability in both INS-1-derived 832/13 cells and primary mouse islets (33). In summary, cytosolic calcium, cAMP, ROS, PKC ζ, ERK1/2, mTOR, and PP2 are key signals for the regulation of glucose-induced Myc expression in β-cells (Fig. 4).

### Hyperglycemia regulates Myc expression in the β-cell *in vivo*

Both major forms of diabetes are defined by chronic elevated blood glucose levels (hyperglycemia) (1, 2). Thus, diabetic conditions necessarily expose β-cells to high glucose concentrations. Moreover, as opposed to many other types of cells, pancreatic islet cells are exposed to comparatively high concentrations of glucose since they are surrounded by a dense network of fenestrated capillaries that allows greater exchange of blood glucose with β-cells (85, 86, 87). Furthermore, at least in rodents, the presence of Glut2, a high-capacity, low-affinity glucose transporter, exposes β-cells to high rates of glucose uptake and metabolism (85, 88). It therefore follows that hyperglycemic conditions might affect the regulation of Myc levels in β-cells *in vivo*. Indeed, various *in vivo* hyperglycemic rodent models display increased Myc levels in pancreatic islets. For example, partial pancreatectomy in rats, in which 85–95% of the pancreas is removed, results in hyperglycemia and more than a fivefold increase in *Myc* mRNA expression in islets (22). By contrast, administration of phlorizin (an inhibitor of the renal sodium-glucose transporter) to these rats restores blood glucose and *Myc* expression to normal levels in islets (22). In another model, rats infused with glucose (500 g/L at flow rate of 2 ml/h) for 24 h display a 3.6-fold increase in plasma glucose concentration (from 5.5. to 20 mM) and a twofold increase in *Myc* mRNA expression in islets. Similarly, when blood glucose is constantly adjusted to 11 mM for 4 days using a glucose clamp, *Myc* mRNA expression in islets increases by twofold compared with the control group (22). Therefore, when β-cell regeneration is induced (pancreatectomy), or when there is an increase in insulin demand (glucose infusion), Myc expression is upregulated in islets. However, whether this upregulation is required for adaptive β-cell proliferation in these two rodent models has not been studied. Taken together, these studies indicate that high glucose levels increase Myc expression in islets *in vitro* and *in vivo*.

Acute high-fat diet (HFD) feeding of young mice leads to hyperglycemia, increased β-cell replication, and enhanced mRNA expression of several Myc target genes in islets (33). However, whereas *Myc* mRNA levels are unchanged, Myc protein levels in islets increase two- to threefold, probably due to increased Myc protein stability induced by Myc Ser62 phosphorylation *via* PKC ζ (33). Concomitant with hyperglycemia, mice fed HFD display increased plasma insulin due to increased insulin demand (7, 8, 33, 89). Therefore, a question arises as to whether increased plasma insulin is another trigger for increasing Myc levels in islets. Jonas *et al*. (74) showed that addition of exogenous (1 μM) insulin to primary rat β-cells does not provoke any changes in Myc expression. Furthermore, they showed that addition of clonidine, an inhibitor of ATP-sensitive potassium channels (90), reduces insulin secretion but does not block the glucose-stimulated increase of *Myc* mRNA (74). Although *Myc* expression is increased in the partial pancreatectomy rat model, it is accompanied with a reduction of insulin gene expression in islets and decreased plasma insulin (22). Thus, the combination of all the *in vitro* and *in vivo* studies mentioned above indicates that glucose, rather than insulin, promotes increased Myc levels during hyperglycemic conditions.

## The two faces of Myc in the pancreatic β-cell

### Sustained and high Myc overexpression induces β-cell death and diabetes

Normal adult β-cells have intrinsically low proliferative rates that correlate with low Myc expression levels (5, 6, 22, 33, 74, 91). As mentioned above, hyperglycemia stimulates the expression of Myc in pancreatic β-cells (22, 74), suggesting a potential role for this transcription factor as an essential part of the adaptive β-cell proliferation machinery. As expected, transgenic mice with sustained overexpression of Myc in β-cells display increased β-cell proliferation. However, this is followed by a rapid onset of β-cell dysfunction (downregulation of insulin expression) and β-cell apoptosis that quickly progresses to diabetes (26, 27, 28, 29). Furthermore, transgenic mice with tamoxifen-inducible overexpression of an active nuclear-restricted form of human Myc under the insulin promoter (pIns-c-MycER^TAM^) in adult β-cells also display both rapid onset of β-cell proliferation and apoptosis leading to diabetes (29), confirming the previous results observed in transgenic mice with sustained Myc overexpression (26, 27, 28). Interestingly, hyperglycemia *per se* does not contribute to Myc-induced β-cell apoptosis since blood glucose normalization by insulin treatment or islet transplantation in these transgenic mice does not prevent nor reduce β-cell loss (29). Gene expression analysis of islets from these mice showed that Myc overexpression leads to activation of DNA-damage checkpoint pathways, stabilization of p53, and activation of proapoptotic-signaling pathways like Cdc2a and p19Arf (29). Myc-induced apoptosis correlates with increased expression of Bax, a proapoptotic Bcl-2 family member that antagonizes the antiapoptotic effect of Bcl-2, demonstrating that Myc overexpression-induced β-cell loss is mediated by an intrinsic mitochondrial apoptotic pathway (92). Taken together, these studies in transgenic mice indicate that sustained and high Myc overexpression in β-cells leads to β-cell death and dysfunction and suggests an important role for Myc in glucotoxicity-induced β-cell demise in chronic hyperglycemia and diabetes. Furthermore, if Myc was once thought to be a useful therapeutic target for β-cell regeneration for the treatment of diabetes because of its capacity to enhance β-cell replication, the β-cell death and dysfunction associated with its overexpression completely eliminated this idea. However, as shown below, the high level and chronicity of expression of Myc in β-cells in these transgenic mice likely explain the triple actions of Myc, inducing proliferation, death, and dysfunction, since mild, acute, transient physiologic upregulation of Myc leads to β-cell proliferation without detrimental effects on β-cell life.

### Myc is required for neonatal and adaptive β-cell replication

Immature β-cells during the early postnatal period undergo functional maturation and acquire the glucose-responsive insulin secretory phenotype (93, 94). This process results in the capacity of the β-cell to adapt its mass and function in order to increase insulin secretion and efficiently control blood glucose in the adulthood (95, 96). These observations raise the questions of which mechanisms trigger β-cell expansion and functional maturation in newly formed β-cells and whether these two β-cell features are mutually exclusive. A recent study by Puri and colleagues indicates that Myc protein abundance is enhanced in juvenile islets in rodents, thus promoting a high proliferation rate in neonatal β-cells (32). Moreover, ablation of Myc in neonatal β-cells leads to decelerated cell cycle progression, compromised proliferation, and reduced functional β-cell mass at postnatal day 16. Indeed, the primary effect of Myc activation in postnatal β-cells appears to be cellular proliferation (32). After the β-cell matures, physiological Myc activity remains at low levels, which is sufficient for the maintenance of β-cell function. To determine whether Myc initiates proliferation of adult β-cells while maintaining a β-cell mature state, Puri and colleagues developed an inducible mouse model where the Myc gene is under control of the insulin promoter. Activation of low levels of Myc leads to increased β-cell proliferation, increased β-cell mass, and a trend toward hypoglycemia (32). No evidence of enhanced β-cell death was observed in these studies suggesting that Myc is required for early postnatal β-cell expansion and that mild upregulation of Myc in β-cells increases β-cell proliferation and mass in adults. However, when this mild upregulation was maintained for a long period of time (1 year), β-cell dedifferentiation occurred possibly by a combination of pro-dedifferentiation actions of chronic Myc activation and the sustained mild hypoglycemia observed.

During pregnancy, adaptive β-cell expansion occurs due to an increase in insulin demand (9, 10). Analysis of pregnancy-induced changes in the islet proteome at the peak of β-cell proliferation in mice (gestational day 14.5) predicts that Myc is one of the main upstream regulators mediating β-cell mass expansion (97). This suggests that Myc might be upregulated in β-cells during pregnancy when maximal proliferation occurs and that Myc might be required for adaptive β-cell proliferation during pregnancy. However, this is currently unknown.

Overnutrition by HFD feeding triggers an early adaptive increase in β-cell proliferation that leads to compensatory β-cell mass expansion to cope with the enhanced insulin demand (7, 8, 98, 99, 100). PKC ζ activity regulates glucose- and acute HFD-induced β-cell proliferation (100). However, how PKC ζ regulates β-cell proliferation in this context of enhanced insulin demand and whether Myc activation could be involved in this process were unknown. RNAseq analysis of islets from young mice acutely fed with HFD for 1 week revealed that most of the significantly upregulated genes were Myc targets and belonged to cell cycle and cell division pathways by gene set enrichment analysis (33). Myc protein expression in islets and β-cells of young mice fed a HFD was increased by two- to threefold. Interestingly, β-cell proliferation and Myc expression induced by HFD feeding were impaired in transgenic mice expressing a kinase dead form of PKC ζ in β-cells, suggesting that Myc could participate in the regulation of adaptive β-cell proliferation downstream of PKC ζ in this context (99) (Fig. 4). This is the case, Myc deficiency in β-cells of young mice fed HFD impairs adaptive β-cell proliferation and mass expansion (33). Furthermore, Myc deficiency in β-cells of these mice leads to impaired glucose tolerance and hypoinsulinemia during overnutrition indicating that Myc is required for the adaptive response of the β-cell during an acute metabolic challenge.

Unlike young mice, 1-year-old mice fed the same HFD display an increase in both Myc expression and stability in β-cells, but do not induce Myc targets. Therefore, HFD increases Myc abundance in islets of young and old mice but impairs Myc action in old mouse β-cells (33).

## “Myc resistance” in the aged β-cell

### Epigenetic regulation of the β-cell in aging

The rate of β-cell proliferation in rodents and humans diminishes dramatically with aging, when β-cell mass expansion stalls, insulin resistance increases, β-cell functionality enhances, and the incidence of hyperglycemia, and eventually T2D, is highly increased (2, 3, 5, 6, 91, 101, 102). Basically, adaptive β-cell expansion response to increased insulin demand is halted with aging.

A large body of evidence has recently shown that the regulators of β-cell homeostasis in normal and adverse metabolic conditions are epigenetic modifications (103, 104, 105, 106, 107, 108, 109, 110, 111, 112, 113, 114, 115). Epigenetics are the environmental influence on gene regulation that could be inherited to the next generation, do not rely on changes in the primary DNA sequence, and dictate how cells respond and adapt to diet, exercise, stress, and circadian rhythms (103, 104). Epigenetic regulation includes DNA methylation, noncoding RNAs (ncRNAs), histone posttranslational modifications, and ATP-dependent chromatin remodeling complexes (105, 106, 107). In this review, we will focus on DNA methylation that occurs primarily on the CpG dinucleotides by the addition of a methyl group on cytosines. This epigenetic mark can have profound impacts on transcriptional repression and cellular phenotype (108, 109, 110, 111, 112). Studies analyzing genome-wide profiles of DNA methylation in human islets from healthy and T2D individuals show specific changes in the islet methylome in diabetes, resulting in the alteration of expression of genes that are critical for insulin secretion, β-cell adaptation, and survival (113, 114, 115).

Large-scale changes in DNA methylation patterns across metabolic tissues reflect the epigenetic regulation underlying insulin resistance that results from overnutrition and obesity. Change in diet composition, such as the lipid content of an HFD, has a dramatic impact on the fat, liver, muscle, and islet epigenomes, especially in genomic regions associated with metabolism (116, 117, 118, 119). However, whether acute HFD feeding and the corresponding changes in the DNA methylome have an impact on the expression of genes required for adaptive β-cell expansion has not been described. Below we summarize the current knowledge on the effect that acute HFD feeding has on islet DNA methylation, Myc DNA binding, and Myc requirement for adaptive β-cell proliferation in young and old mice.

### Myc upregulation in the metabolically stressed aged β-cell: epigenetically mediated “Myc resistance”

Although aging restricts β-cell proliferative capacity, mild (two- to threefold) Myc upregulation robustly and equally induces β-cell proliferation in islets from 8-week-old and 1-year-old mice (33). Therefore, there is no impairment of Myc action on β-cell proliferation in aging mice, an aspect previously observed in adult human β-cells (30). As mentioned before, 1-week HFD feeding in young mice leads to hyperglycemia, hyperinsulinemia, cell cycle activation, Myc upregulation and nuclear localization in β-cells, and Myc-dependent adaptive β-cell proliferation (33). The same diet administered for 1 week to 1-year-old mice leads to hyperglycemia, hyperinsulinemia, β-cell Myc upregulation, and nuclear localization but no adaptive β-cell proliferation (33). This discrepancy between the identical expression of Myc and the absence of adaptive β-cell proliferation in aged mice fed with HFD can only be explained by different adaptation of the β-cell to the HFD feeding since β-cells from 1-year-old mice fed a regular diet are capable of responding to Myc upregulation by increasing β-cell replication similarly to β-cells from young mice. These experiments also suggest that aging *per se* is not responsible for the impairment of Myc action observed with HFD in aged mice.

RNAseq analysis of the islet transcriptome clearly identified a completely different set of upregulated genes by 1-week HFD feeding in young and aged mice. In young mice, upregulated genes correspond to cell cycle or cell division pathways; however, in aged mice, upregulated genes were not associated with cell cycle or cell proliferation biological processes, and Myc failed to bind to E-boxes of cell cycle gene promoters (33). This indicates that HFD feeding increases Myc expression and nuclear localization in β-cells and favors binding of Myc to cell cycle promoters in β-cells of young mice, but this access to cell cycle gene promoters is not present in the β-cell of aged mice.

**Figure 5:**
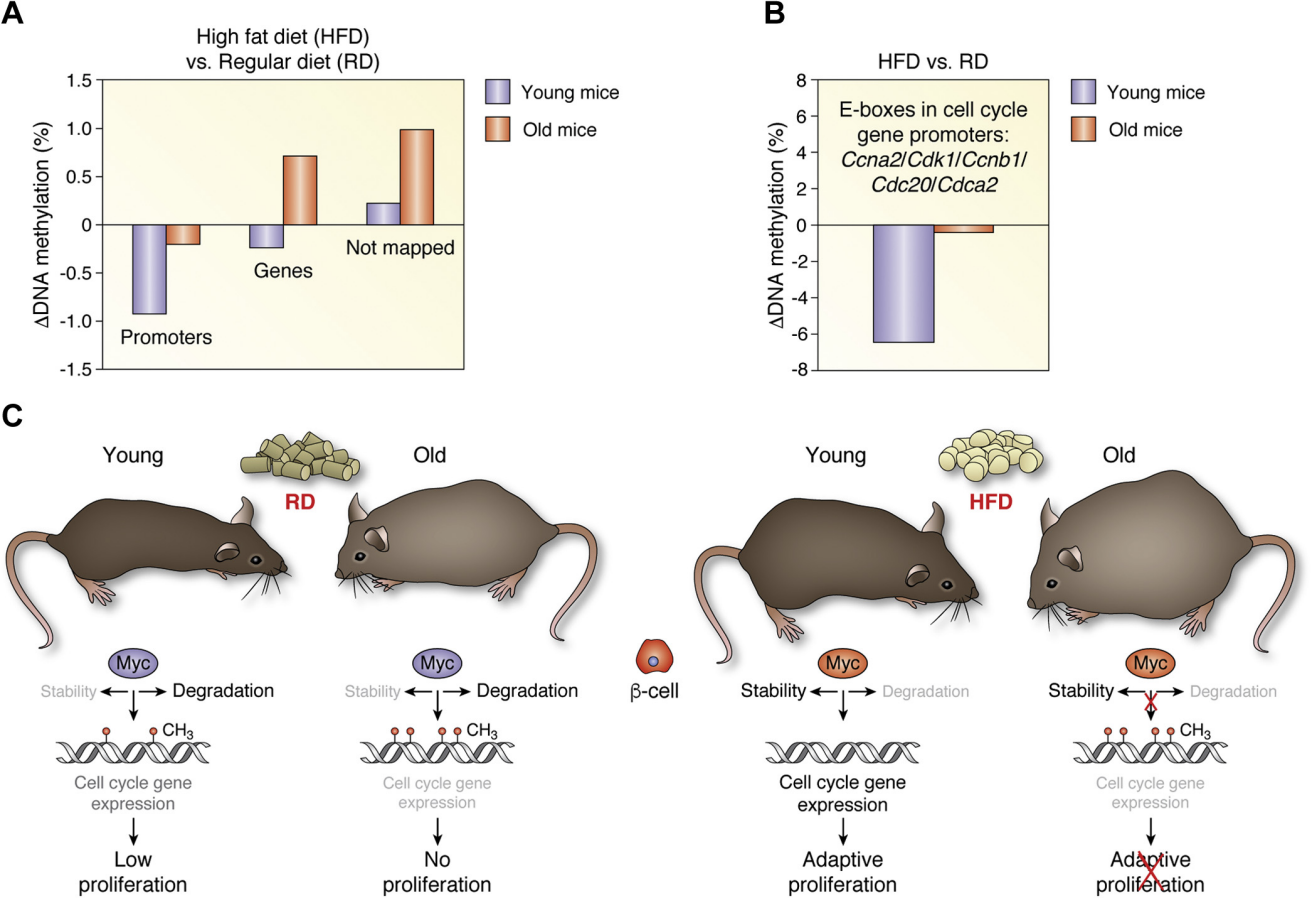
**Adaptive β-cell proliferation, Myc stability and action, and DNA methylation in young and aged mice under metabolic stress.***A* and *B*, methylation of E-boxes in promoters of cell cycle genes in β-cells from young and aged mice fed regular diet (RD) or high-fat diet (HFD). Differential methylation across all CpG dinucleotides of a region spanning a total of about 1 Mbp containing upregulated cell cycle regulatory genes by HFD in islets from young mice. DNA methylation for CpG dinucleotides was averaged in each group and then subtracted as follows: 8-week-old mice fed HFD—8-week-old mice fed RD (*black*); 1-year-old mice fed HFD—1-year-old mice fed RD (*white*). Differential methylation in (*A*) promoters, gene bodies, and not otherwise mapped regions in the target region and (*B*) in E-boxes of promoters of specific cell cycle genes after 1-week HFD in young and old mice. Adapted from Rosselot *et al*. Myc is required for adaptive β-cell replication in young mice but is not sufficient in 1-year-old mice fed with a HFD. *Diabetes* 2019; 68: 1934–1949. Copyright 2019 by the American Diabetes Association. *C*, summary of the effects of diet and age on Myc stability and action and β-cell proliferation. β-cells in young mice fed a RD (upper left) display low levels of Myc and low β-cell proliferation rates. β-cells in aged mice fed a RD (upper right) display very low levels of Myc and β-cell proliferation is almost absent. After HFD feeding, β-cells in young mice (lower left) display increased Myc stability leading to high levels of Myc expression, which is required for cell cycle gene expression and adaptive β-cell proliferation and function. β-cells in aged mice fed a HFD (lower right) display increased Myc stability leading to high levels of Myc expression, but compromised binding to cell cycle gene promoters due to the absence of HFD-induced DNA demethylation. The end result is that adaptive β-cell proliferation is impaired in aged mice.

HFD feeding can lead to epigenetic modifications, and indeed HFD induces global DNA hypomethylation in the liver and adipose tissue of young rodents when compared with rodents fed a regular diet (118, 120). Thus, it could be possible that the DNA methylome is different in young and aged β-cells from mice fed HFD. Indeed, analysis of DNA methylome sequence of the CpGs in regulatory regions of 21 Myc target cell cycle genes in β-cells at both ages uncovered that E-boxes were heavily demethylated in β-cells from young mice fed HFD for 1 week compared with young mice fed regular diet (33). This suggests that acute HFD feeding favors DNA demethylation and DNA binding of the upregulated Myc to E-boxes in young β-cells. However, DNA demethylation was not observed in E-boxes in cell cycle genes of β-cells of aged mice fed HFD. This indicates that HFD-induced DNA-demethylation is impaired in aged β-cells (Fig. 5). Interestingly, treatment of mouse islets from aged mice fed HFD with the inhibitor of DNA methylation 5-aza-2′-deoxycytidine decreases global DNA methylation, partially rescues the binding of Myc to promoter regions of cell cycle genes, and induces mild β-cell proliferation in these islets. This increase in β-cell proliferation is dependent on Myc action since the Myc inhibitor 10058-F4 completely abolished β-cell proliferation induced by 5-aza-2′-deoxycytidine treatment (33). These studies suggest that other epigenetic modifications such as acetylation or phosphorylation, or changes in the presence/expression of long-noncoding RNAs such as Pvt-1, microRNAs, DNA methylases/demethylases, the Myc-binding partner Max, or regulators such as Mad, Mxg, Mga, or Mnt might also play a role in the absence of Myc action and compensatory β-cell replication and expansion in aging. Whether alleviating this “Myc resistance” in specific Myc target genes could lead to adaptive compensation to aging, and in that manner halt the progression to hyperglycemia and diabetes, would be an important hypothesis to be tested.

## Myc as a target for β-cell regeneration: the story of harmine

The progressive loss of β-cell mass and function, which contributes to a reduction in insulin secretion, leads to T1D and T2D. One area of diabetes research focuses on finding new approaches to regenerate sufficient endogenous insulin-secreting β-cells for optimal blood glucose regulation (3, 4). Human β-cells proliferate after birth at a rate of about 1–3%, but proliferation rates decline rapidly with age and remain low (∼0.01%) for the rest of adulthood (91, 101, 121). While β-cells can be coaxed to replicate using gene therapy approaches to increase cyclin and kinase components of the cell cycle machinery (122, 123, 124, 125, 126), this is not an attractive therapeutic approach, and much work needs to be done to understand intracellular pathways linked to cell cycle regulation and β-cell proliferation and survival. One potential therapeutic strategy is the discovery of small molecules capable of expanding β-cell mass that would provide enormous benefit for the large population of patients with diabetes. Based on the evidence that mild Myc activation leads to enhanced β-cell replication and mass without alteration in β-cell function, Wang *et al*. (31) performed a high-throughput screening of more than 102,000 compounds from two small molecule libraries for their capability to activate the human *MYC* promoter using the human hepatocyte cell line HepG2. Among these compounds, the authors identified harmine as an alkaloid capable of both mildly upregulating Myc expression in human islets and robustly increasing BrdU incorporation and Ki67 immunolabeling in dispersed rat and human pancreatic β-cells, while avoiding both DNA damage and β-cell apoptosis. Additionally, harmine-treated human islets display increased *INS* mRNA expression that correlates with higher expression levels of known regulators of β-cell function including *PDX1*, *NKX6.1*, and *MAFA* (31).

Harmine is a competitive inhibitor of the dual-specificity tyrosine phosphorylation-regulated kinase (DYRK) 1A, but it can inhibit other DYRK family members, monoamine oxidases (MAOs), and cdc-like kinases (CLKs) (127). Wang and colleagues demonstrated that the mitogenic effect of DYRK1A inhibitors operate through NFAT; dephosphorylation of NFAT by the phosphatase calcineurin allows its translocation to the nucleus and subsequent target gene expression (128, 129). NFAT binds the promoters of cell cycle genes and stimulates the expression of cyclins A2 and D2 (*CCNA2*, *CCND2*) and cdk 1 (*CDK1*), while decreasing the expression of Cdk inhibitors such as *p15INK4*, *p21CIP*, and *p57KIP2* (31, 130, 131, 132, 133). Nuclear NFATs are then phosphorylated by glycogen synthase kinase-3 (GSK3), casein kinase 1 (CKI), and DYRK1A, promoting translocation back into the cytoplasm (128, 129, 130, 134). It is important to note that 10058-F4 (a Myc inhibitor) effectively blocks harmine-induced human β-cell proliferation (31). Using a different approach, Cre-mediated excision of *Myc* from islets of Myc^loxP/loxP^ mice decreases harmine-induced β-cell proliferation (31). Thus, harmine-stimulated β-cell proliferation requires Myc. More recently, combination of harmine with TGFß inhibitors or GLP-1R agonists has been shown to induce a striking increase in human β-cell proliferation (5–8%), suggesting that combination therapies affecting several signaling pathways could further enhance human β-cell regeneration for diabetes treatment (135, 136). Collectively, these results indicate first that a mild increase in Myc expression with harmine leads to robust human β-cell proliferation similar to the levels found in early postnatal ages (1–2%); second, that combination of harmine with other modulators of intracellular signaling can lead to β-cell proliferation rates beyond the levels found in postnatal ages; and third, that the robust increase in β-cell proliferation is accompanied by an improvement in the expression of β-cell functional markers highlighting the potential therapeutic future of harmine for β-cell regeneration once means are found to target this small molecule to the β-cell.

## From harmful to necessary: Myc effects on pancreatic β-cell function

Supra-physiological expression of Myc in rat islets decreases insulin expression and reduces glucose-stimulated insulin secretion (GSIS) (137). Similarly, islets isolated from transgenic mice overexpressing Myc in the β-cell display mitochondrial membrane hyperpolarization, defective glucose-induced calcium release, and inhibition of GSIS (138). Furthermore, acute activation of Myc in pIns-c-MycER^TAM^ transgenic mice initially results in highly increased serum insulin levels and subsequent hypoglycemia, whereas chronic activation of Myc in these mice leads to a significant decrease in serum insulin, resulting in hyperglycemia (29). Interestingly, human β-cells that mildly overexpress Myc or that are treated with harmine, a mild pharmacological Myc agonist do not display any changes in GSIS compared with control (30, 31). These discrepancies in the effect of Myc overexpression in β-cell function could be related to the level or duration of expression of this transcription factor.

Interestingly, 3-month-old transgenic mice with mild overexpression of Myc in β-cells display enhanced β-cell proliferation and mass, hypoglycemia, improved glucose tolerance, and normal insulin content per β-cell suggesting that at least 3 months of mild Myc overexpression in β-cells has beneficial effects in terms of β-cell expansion and function (32). However, islets from these mice display imprecise glucose sensing, significantly lower insulin secretion indices but normal GLP-1-induced insulin secretion. Moreover, islets from these mice display increased proinsulin levels, decreased prohormone convertase PC1/3, and a gene profile characteristic of β-cell immaturity. It is important to note that while β-cell immaturity increases over time in these Myc ovexpressing mice, glucose tolerance impressively improves with aging suggesting that the remarkable increase in β-cell mass in these mice is sufficient to provide beneficial effects on controlling glucose homeostasis even in the context of decreased β-cell maturity and aging. Whether the inappropriate GSIS and decreased β-cell maturation in these mice are the result of Myc itself or an adaptive response to markedly enhanced β-cell mass and chronic hyperinsulinemia is unknown.

Overexpression of proteins above normal physiological levels may lead to cellular damage that can hinder the ability to characterize the true physiological role of the protein (139). To determine the physiological importance of Myc for β-cell function *in vivo*, glucose homeostasis was measured in mice with inducible deletion of Myc in β-cells (32, 33). Adult mice with deleted Myc in β-cells showed normal blood glucose levels, plasma insulin, and glucose tolerance in basal conditions suggesting that Myc is not required for the function of the adult β-cell in normal conditions. However, when these mice were fed with a HFD for 1 week, they displayed hyperglycemia, hypoinsulinemia, and impaired glucose tolerance, indicating that in situations of metabolic stress Myc expression is required for proper β-cell function (33). This clearly contrasts with the studies described above regarding overexpression of Myc in β-cells in transgenic mice. Therefore, whereas too much Myc is harmful for the β-cell, low physiological levels of Myc are required for maintaining normal β-cell function (Fig. 2).

Alterations in mitochondrial function lead to inefficient GSIS in β-cells (140). Myc regulates numerous metabolic processes including glucose and glutamine metabolism and mitochondrial biogenesis (21, 141, 142). However, whether Myc controls glucose metabolism and mitochondrial function in β-cells exposed to overnutrition is completely unknown. Importantly, preliminary studies suggest that Myc action in β-cells is required for 1) glucose-induced enhancement of mitochondrial membrane potential; 2) ATP production induced by glucose; and 3) efficient glycolysis and mitochondrial metabolism (Scott *et al*. unpublished observations).

Interestingly, like in cancer cells (49, 143, 144), Myc may direct metabolism in β-cells in favor of metabolic pathways that support β-cell proliferation. According to these studies, Myc upregulates the expression of genes associated with RNA metabolism, protein metabolism, ribosome biogenesis, and ribosome function (32). Additionally, Myc controls the expression of selenocysteine biosynthesis, ornithine decarboxylase, and lactate dehydrogenase A, all of which promote cell proliferation in other cell types (144, 145, 146). Future studies are needed to further analyze how Myc influences β-cell metabolism in order to obtain a better understanding of the mechanisms by which Myc supports the adaptive increase of β-cell mass and function.

## Conclusions and perspectives

Myc has gone through several research life phases in the β-cell since the 1980s. Initial studies indicated that Myc is upregulated in β-cells exposed to high glucose levels, highlighting its potential importance for diabetes. Most of the studies that followed used transgenic, transfection, or infection approaches to deliver Myc in rodent β-cells and concluded that Myc overexpression is detrimental to the function and life of the β-cell, dropping the interest for this molecule as a target for potential therapeutic intervention in regenerative therapies for diabetes. However, in the last 5 years, new experimental evidence has concluded that mild Myc overexpression induced by small molecules such as harmine can lead to impressive increases in adult human β-cell proliferation. Whether this translates to increases in actual β-cell mass *in vivo* in human islet xenografts is unknown. Also, whether harmine can be targeted to the β-cell to eliminate potential side effects in other tissues has not been achieved yet. Studies in these directions are warranted.

The generation and characterization of β-cell specific Myc knockout mice in the last 3 years have also switched the scientific thought from Myc expression being detrimental for the β-cell in terms of function and survival to the necessity of physiologic upregulation of Myc in the β-cell for postnatal β-cell proliferation and adaptive β-cell replication. However, many aspects of the potential physiological role of Myc on the regulation of the β-cell function have not yet been uncovered. Furthermore, if future experimental approaches can relax the “Myc resistance” present in the metabolically stressed aged β-cell, it could bring therapeutic optimism to the aging population more prone to insulin resistance and T2D. In summary, studies in the literature suggest that the time and the dose explain how the “villain” Myc can turn into the “hero” Myc in the β-cell. More studies are needed to truly unravel the fascinating biology of this not-that-well-known transcription factor in the β-cell.

## Conflict of interest

The authors declare that they have no conflicts of interest with the contents of this article.
